# 

**Published:** 1813-06

**Authors:** 


### MONTHLY CATALOGUE OF MEDICAL BOOKS.

ELEMENTS of Agricultural Chemistry, in a Course of Lectures for the Board of Agriculture. By Sir Humphry Davy, L.L.D. 4to.—Longman and Co.

Researches about Atmospheric Phenomena, together with Meteorological Journals, &c. By Thomas Forster, F.L.S. 8vo.—Underwood.

A Synopsis of the various Kinds of Difficult Parturition. By Samuel Merriman, M.D. 12mo.—Callow.

A Treatise on the Management of Parturient Animals. In Two Parts. By T. Parkinson, M.D. 12mo.—Callow.

Elements of Crystallography, after the Method of Haüy. By Frederick Accum. 8vo.—Longman and Co.

### *Books in Hand, or in the Press.*

Mr. I. M. Coley, of Bridgenorth, is about to publish a Practical Treatise on the Remittent Fever of Infants, with remarks on several other diseases, particularly Hydrocephalus Internus.

Mr. Longmire, of Troutbeck, near Kendal, is writing an Essay on Geognosy.

---

### *NOTICES TO CORRESPONDENTS.*

*The Plate published with our last Number* (171), *unaccountably and negligently left without a title by the Engraver, represents the Case of* Cynanche Laryngea, *by Mr. Shillito, published in No.* 168, *p.* 125, *under the title of* "*An inflammatory Affection of the Trachea.*" *The Plate is intended to face page* 125.

*We have received a letter from Mr Saumarez, requesting us to state, that subsequent experiments have occasioned some doubts in his mind respecting the accuracy of the one contained in p.* 87 *of his Oration; an imperfection having been since discovered in the apparatus, but which does not, in any way, affect any of the former experiments, or the deductions which he has made from them.*

*The text, page* 283, *stating that* ℥j *of peppermint-water was given three times a-day, is correct.*

*We regret that a letter from a medical gentleman at Bedford has been mislaid. It contained, we believe, some remarks upon Dr. Yeats's paper on Ischuria. Should the author oblige us with another copy, we request he will enter more into detail, for we doubt whether such a brief statement as the former one would be sufficiently interesting for publication.*

*We have received communications from Dr. Kinglake; Dr. Walker; Mr. Spark; J. W. C.; &c. &c.*

### INDEX

TO THE TWENTY-NINTH VOLUME.

APOTHECARIES and surgeon-apothecaries, observations on their projected reform 2, 3, 4, 5, 6, 7, 8, 9
Arsenic, on its medical use 12
Apoplexy, remarks on 15
Arsenic, observations on poisoning with 44
———, Hume on a test for 46
ADAMS, Mr. on the origin of one of his instruments, and operations for the cure of cataract 89
*Ava* root, inquiry concerning 108
Apothecaries and physicians, on the rights of 277
Analogies between vegetables and animals 298
Apothecaries act, impolicy of 301
Adams, Mr. new mode of treating Egyptian ophthalmia by emetics 302
Address of the committee of apothecaries, &c. 346
Apothecaries, committee of, their meeting with the country deputies 349
———, education of 393
———, defence of 394
——— act, utility of 396
Air, factitious, experiments on, account of 399
———, Cavendish's papers on 404
Astronomy, Mr. Cavendish's papers on 407
Atropa Belladona, history of its employment for dilating the pupil 422
Animal heat, on the production of 441
——— depends on respiration 443
Antimony, use of, in ophthalmia 452
Apothecaries, strictures on their bill 456
Animals increase and decrease, laws of nature respecting 502-504
Alcohol of sulphur, Dr. Marcet on 509
Arragonite, analysis of 510
Animals, observations on, when exposed to high temperature 511
Analysis of vegetables, by Vauquelin 519
Botany and materia medica, progress of 10, 12
Bucknill, Mr. on poisoning by arsenic 42
BOOKS, NEW MEDICAL, &C. CRITICAL ANALYSIS OF.—New England Journal of Medicine and Surgery, &c. No. ii. 50. Edinburgh Medical and Surgical Journal, No. xxxi. 63. Parkinson, Dr. T. Manual of the Treatment of parturient Animals, 66. Mr. Maiden's Case of recovery from a Wound through the Thorax, 68. Dr. Christie on Small-pox and Vaccination in Ceylon, 72, 155. Dr. Farre's Morbid Anatomy of the Liver, Part I. 152. Home and Foot on the Prostate Gland, 226. Adams's, Mr. Practical Observations on Diseases of the Eye, 312. New-England Journal of Medicine and Surgery, No. iii. 328. Dr. Bradley's Treatise on Worms, 334. Chamberlaine on Dolichos Pruriens, 335. Dr. Henderson's Examination of the Imposture of Ann Moore, 408. Edinburgh Medical and Surgical Journal, No. xxxii. 402. Stevenson's Treatise on Cataract, 420. Dr. Merriman's Synopsis of difficult Parturition, 423. Cases of Hydrophobia by J. O'Donnel, M. D. 485. Bosworth's Accidents of Human Life, 492. Chamberlaine's Tirocinium Medicum, 494. Saumarez's, Mr. Oration before the Fellows of the Medical Society of London, 497. Fothergill's Essay on Natural History, 501
Books announced 84, 174
BURNS, on the application of cotton in 137

Brain, on its influence in the generation of animal heat, by Mr. Brodie (with a plate) 141
Burns, on Dr. Kentish's method of treating, with a case 196
Books, catalogue of, employed in the private medical school at New York 253
Botanical report 260, 433
Ball, wind of a, remarks on its cause of death 63
Brown, Mr. cases of small-pox after vaccination 281
Barlow, Mr. James, case of morbid tumor on the nose 296
Buxton, Dr. cases of pulmonic disease 376-391
Biography of the Hon. H. Cavendish 397-407
Brain, influence of, in the production of animal heat, examination of Mr. Brodie's opinions on 441
Bladder, inflammation of, on bleeding in 455
Brain, case of concussion of 460
Birds, description of the eye of 481
Brentford, fossils found in the neighbourhood of 507
Committee, London, of apothecaries, observations on 2, 3, 4, 5, 6, 7, 8, 9, 79, 258
Chemistry, progress of, from July to December 1812 9
Chemical controversy between Hume and Marcet 10
Collectanea Medica 48, 141, 304, 397, 474
Catalogue, monthly, of books 88, 176, 263, 351, 438, 528
Cataract, Mr. Adams's operations for 89
Christie, Dr. on blood-letting in hydrophobia 105
*Chopperpoora*, poisonous snake of India, symptoms of its bite 123
Cynanche laryngea, case of, by Mr. Shillito (with a plate) 125
Committee of apothecaries, letter to, by Mr. Parkinson 132
Cotton, cases of its application to Burns 137
Cases—Of hydrophobia, 24; of aneurism of the aorta, 53; of disease of the lungs, &c. 55; of necrosis, 60; of wound in the thorax, 68; of cynanche laryngea, 125; of cotton in burns, 137; of caries ossium cranii ex syphilitide, 178; of diseased spine, 183, 189; of burn, 196; of *Isaac Casaubon*, 245; extirpation of diseased tonsils, 265; of croup, 268; of small-pox after vaccination, 281; of cured hydrophobia, 285; of gout relieved by humulus, 294; of morbid tumor on the nose, with a plate, 296; of cataract, 324; of the American spotted fever, 328; of ulcerated uterus, 333; of urine discharged from the stomach and intestines, 363, 365; hydrothorax, successful, of, 369; of pulmonic disease, 376, 391; of tumor on the nose, 391; of pseudo syphilis, 417; of purpura hæmorrhagia, 419; extra-uterine pregnancy, 448; concussion of the brain, 460; of hydrophobia, 485, 489.
Committee of apothecaries, address of 165
——————, outlines of their proposed plan 167
——————, list of subscribers to 168, 258, 340
Caustic, actual, observations on Richerand's recommendation of 181
Chorea sancti viti, observations on, by Dr. Reeve 64
Croup, case of, by Mr. Cornish 268
Cataract, observations on and treatment of 320
Chester, medical topography of, by Dr. Pigot 374
Catheter, elastic gum, on the use of 375
Cavendish, Hon. Henry, biography of 397-407
——————, his chemical papers 399
—————— electrical papers 406
—————— meteorological papers ib.
—————— astronomical papers 407
Cold, extreme, at Hudson's Bay 404
Chorea sancti viti, Dr. Uwins on 414
Cataract, practical treatise on 420
————, symptoms of ib.
————, modes of treating 421
Cuvier, M. on fossil bones 520
Diet, rules for, by Suvorof 49
Dentition, on the morbid effects of 51
Derby, meteorological table for, by Mr. Stanwick 292
Deaths by small-pox 525
Eau Medicinale, D. Menuret's fact respecting 22
Education, medical observations on 118, 275, 288, 445

Ectropium, observations on 313
————, operation for, by Mr. Adams 314
Eudiometer of Fontana, its accuracy proved 403
Electrical papers of Mr. Cavendish 406
Engelbrecht, Hans, his enthusiasm 408
Eruption of the Suffriere volcano in St. Vincent 426
Ectropium, Mr. Adams's new operation for 432
Education, reply to Mr. Jones on 467
Eye of birds, description of that organ in, by Philip Crampton, Esq. 481
Fever, spotted, of America, account of 14, 328
Freake, Mr. on the humulus in gout 294
Fever, spotted, of America, symptoms of 381
Freezing, experiments on, by Cavendish 404
Fontana's eudiometer, its accuracy proved 408
Fever, yellow, on the treatment of 412
————————, at New York in 1803 416
Freezing of alcohol 481
Fire, chemical preparations for extinguishing 493
Fat, on the formation of, by the lower intestines 507
Fungin, analysis and account of 520
Gold, Chrestien's preparation of, used in America 12
Granville, Dr. case of caries ossium cranii ex syphilitide, by 177
Gamage, Dr. on carbonate of iron in ulcerated uterus 332
Gum, liquid, from Botany Bay, chemical account of, by Dr. Thomson 474
Grasses, cotylidons of, remarks on 510
Gases, on the production of and various results from 515
Hydrophobia, effects of bleeding in 12, 23, 105
————————, Dr. Shoolbred's case of 23-42
————————, observations on the treatment of 37
————————, Dr. Kinglake on bleeding in 43
Hume, Mr. observations on Dr. Roget's reply 46
Head, physical condition, its effects on the mind 48
Hernia, comparative statement of 83
Henderson, Dr. case of Ann Moore 109
Heat, on the generation of animal, by Mr. Brodie 141
Hossack, Dr. David, classification of diseases 250
Heart, on the organic diseases of 53
Hydrophobia and tetanus, observations on 272
————————, case of, cured, by Mr. Wynne 285
Humulus, remarks on its use in gout 294
Hydrothorax, case of, successfully treated 369
Heat, animal, on the production of 441
Hydrophobia and gastritis, analogy between 454
Heat, on the quantity of, produced by combustion 512
INTELLIGENCE, medical and philosophical 78, 165, 247, 336, 426, 506
Issues, caustic, in affections of the spine, dissertation on, by Mr. Hill 182
Iron, carbonate of, in ulcerated uterus 332
Ischuria, dissertation on, by Dr. Yeats 353
————, species of ib.
————, dissection of, cases of, by Tulpius 354
———— where the urine is found in various cavities of the body 355
———————————— accounted for by absorption 355-359
———— renalis, remarks on 367
————, observations on coma in 368
Institute, imperial, of France 511
Jackson, Dr. on the morbid effects of dentition 51
Kinken, Anna Maria, a parallel between her case and Ann Moore's 409
LECTURES—Mr. Taunton's on Anatomy, 85. Mr. Brooks' on Anatomy, ib. Dr. Buxton's, Dr. Squire's, Dr. Merriman's, Mr. Shute's, Dr. Adams's, Dr. Clutterbuck's, Dr. Ramsbotham's, Dr. Clarke's, 86. Dr. George Pearson's, Dr. Reid's, Dr. Hooper's, 174. At Surgeons' Hall, 257. Dr. Squire's on Midwifery, 258. Dr. Merriman, 349. Dr. Reid's, Dr. Adams's, Mr. Taunton's, Mr. Chevalier's, Dr. Clutterbuck's, 433. Drs. Hooper and Ager, Thomson on Botany, Squire, Ramsbotham, and Carpue, 526.
Laws respecting physicians, surgeons, and apothecaries, history of 306-311
Lettsom, Dr. lecture on the natural and medical history of tea 336-339
Labors, on the management of 425
Light, on some new properties of 426

Lungs, on the black matter in, by Dr. Pearson 506
Leaves, remarkable change in the taste at different periods of the day and night 511
Lead, new salts of 523
MEDICAL REPORT, half-yearly 1
Medical reform, observations on 2, 3, 4, 5, 6, 7, 8, 9
METEOROLOGICAL TABLE, &c. 87, 175, 263, 350, 439, 527
MOORE, Ann, the fasting woman, observations on her case, by Dr. Henderson 109-118
Millman, Sir Francis, letter to, by Censorinus 129
Mercury, Dr. Francis's dissertation on 207-226
——, detail of its effects when taken into the system 211
——, its use in fevers 213
——, utility in yellow fever doubtful 215
——, remarks on its employment in syphilis 219
——, as *murias hydrargyri*, history of its use 223
Martin, Dr. on necrosis 60
Meteorological table for Sidmouth, by Dr. Clarke 291
—— for Derby 292
Mercury, congelation of 403
Meteorological papers of Mr. Cavendish 406
—— instruments used at the Royal Society house, account of ib.
Moore, Ann, examination of her imposture by Dr. Henderson 408
——, parallel between, and Anna Maria Kinker 409
Munsford, Mr. on animal heat 441
Moore, Ann, account of her confession 469
Meteorological observations at Tottenham 524
Marshall, Dr. death of 425
Necrosis, observations on, with cases 60
Nottingham and Sidmouth, comparative temperature of 293
Notice to correspondents on the apothecaries' act 352, 440
Narwal, on the tusk of, by Home 427
Notices to correspondents 528
Opium, table of the quantity of, given in a case of tetanus 102
Office and duties of the surgeon-apothecary 279
Ophthalmia, Egyptian, mode of treating by emetics 302
Ochil Hills, mineralogical description of 429
Poisons, mineral, on their entering the circulation, by Mr. Davies 133
Patent for refining sugar 249
Physicians and apothecaries, on the rights of 277
*Psylli*, their means of security from the bites of serpents 304
Pupil obliterated, observations on 316
——, artificial, operations for 318
Purpura hæmorrhagia, case of 419
Plants, mono cotyledonous, physiology of 430
Pregnancy, extra-uterine, case of 448
——, dissection of 450
Pulley, Mr. observations on Dr. Yeats on ischuria 461
Paralysis, remarks on the case of, by Mr. Harrold 462
Patents, new 525
Quere respecting bleeding in retention of urine 271
Report, medical, half-yearly (nota) 1
Reform, medical, observations on 2, 3, 4, 5, 6, 7, 8, 9, 129, 201, 206, 306
Rhododendrum chrysanthum in gout 16
Report of the committee of apothecaries 78
—— observations on 79
Root, poisonous, among gentian, account of 107
——, *ava*, inquiry concerning 108
Report, botanical 260
Reeve, Dr. on chorea sancti viti 64
Ramsden, Mr. death and biographical notice of 339
Rathbone-place water, calcareous matter in 402
Roxburgh, Dr. account of his "descriptions of several of the monandrous plants of India, belonging to the Scitamineæ of Linnæus, the Cannæ of Jussieu, and the Drimyrhizæ of Ventenat" 433-438
Remarks on Mr. Harrold's case of paralysis 462
Richmond, Mr. account of the confession of *Ann Moore* 469
Rumford, Count, on the quantity of heat produced by combustion 512
Speer, Dr. C. on water 17-22
Society, philosophical, of London, proceedings of 81, 336
Small-pox, deaths by 83, 257, 349
Snake, observations on the bite of a poisonous 120
——, symptoms of 121
——, cured by spir. ammon. comp. 122
Society, Royal, meeting of 172, 426

Society, Kirwanian, of Dublin 173
———, Linnæan 174, 428, 509
Stevenson, Mr. reply to Dr. Farre 199
Society, Geological, report of 247
School, private medical, at New York, plan of study at 253
Surgeons, Royal College of, lectures at 257
Surgeon-apothecary, on the office of 279
Small-pox, cases of, after vaccination 281
Studies, medical, observations on 200
Sidmouth, meteorological table for 291
——— and Nottingham, comparative temperature of 293
Serpent charmers, how they are secured from the bites of serpents 304
Speculum, Pellier's, remarks on 322
———, new 323
Society, Medical, of London, anniversary of, with the election of its officers, &c. 339
———, Medical and Chirurgical, of London, anniversary of, &c. ib.
Subscribers to the community of apothecaries, &c. 168, 258, 340
Surgeon-apothecary, defence of 395
Spine, curved, on the cure of 415
Syphilis, pseudo, remarks on 417
Society, Wernerian Natural History 429
———, Western Medical, at Plymouth Dock, institution of 432
Sight, short, observations on 426
Sarcocol, observations on 431
Saumarez, Mr. refutation of some dogmas of M. Richerand 498
Society, Royal, meeting of 506
Skin, observations on the color of, by Dr. Wells 508
Seeds, peculiarity in the structure 509
Tetanus, case of, with a table of the quantity of opium given in, by Mr. Leath 100
TOPOGRAPHY, *Medical*, address to the faculty on 264
Tonsils, case of extirpation of, by Mr. Smith 265
Tetanus and hydrophobia, remarks on 272
Temperature, comparative statement of, at Sidmouth and Nottingham 293
———, general remarks on, and the mode of ascertaining ib.
Tumors, remarks on the classification of 297
Tea, natural and medical history of 336-339
Topography, medical, of Chester 374
Temperature, regulated, its use in pulmonic disease, with cases, by Dr. Buxton 376-391
Tumor on the nose, case of, by Mr. Davies 391
Tetanus, opium and the warm bath in 415
Tumor, subcutaneous, observations on 418
Testis, peculiar disease of 419
Tucker, Mr. case of extra-uterine pregnancy 448
Vaccine disease, laws of, in India 163
Ulcers, remarks on the state of the bowels in 200
Urine, retention of, quere respecting bleeding in 271
Verbal error respecting Messrs. Barlows corrected 289
Vegetables and animals, analogy between 298
Uterus, ulcerated, carbonate of iron in 322
Urine, discharges of, by the skin, histories of 359
——— intestines 361
——— stomach 362
Urine, cases of discharge from the stomach and intestines 363, 365
Vegetables in minerals 430
Venæsection in inflammation of the bladder 445
Water, inquiry into the physical constitution of 17
——— considered as a fluid ib.
——— solid 19
——— gas 20
Warren, Dr. on the organic diseases of the heart 53
———, on the organic diseases of the lungs 55
Weatherhead, Mr. on bleeding in retention of urine 271
Wynne, Mr. case of cured hydrophobia 285
Weather, comparative temperature of, at Sidmouth and Nottingham 293
Worms, symptoms of 334
Waters, mineral, analysis of 402
Water of Rathbone-place, earthy matter in ib.
Wire, on the drawing fine 428
Yeats, Dr. on ischuria 353

### PLATES.

Case of Cynanche Laryngea - - - to face page 125
Mr. Brodie's Apparatus - - - - - - - 142
Case of Morbid Tumor of the Nose - - - - 296

